# Heterogenous ribonucleoprotein A18 (hnRNP A18) promotes tumor growth by increasing protein translation of selected transcripts in cancer cells

**DOI:** 10.18632/oncotarget.7020

**Published:** 2016-01-25

**Authors:** Elizabeth T. Chang, Palak R. Parekh, Qingyuan Yang, Duc M. Nguyen, France Carrier

**Affiliations:** ^1^ Marlene and Stewart Greenebaum Cancer Center, School of Medicine, Department of Radiation Oncology, University of Maryland, Baltimore, MD, USA

**Keywords:** cancer, hnRNP A18, hypoxia, protein translation, RNA-binding protein

## Abstract

The heterogenous ribonucleoprotein A18 (hnRNP A18) promotes tumor growth by coordinating the translation of selected transcripts associated with proliferation and survival. hnRNP A18 binds to and stabilizes the transcripts of pro-survival genes harboring its RNA signature motif in their 3′UTRs. hnRNP A18 binds to ATR, RPA, TRX, HIF-1α and several protein translation factor mRNAs on polysomes and increases *de novo* protein translation under cellular stress. Most importantly, down regulation of hnRNP A18 decreases proliferation, invasion and migration in addition to significantly reducing tumor growth in two mouse xenograft models, melanoma and breast cancer. Moreover, tissue microarrays performed on human melanoma, prostate, breast and colon cancer indicate that hnRNP A18 is over expressed in 40 to 60% of these malignant tissue as compared to normal adjacent tissue. Immunohistochemistry data indicate that hnRNP A18 is over expressed in the stroma and hypoxic areas of human tumors. These data thus indicate that hnRNP A18 can promote tumor growth in *in vivo* models by coordinating the translation of pro-survival transcripts to support the demands of proliferating cells and increase survival under cellular stress. hnRNP A18 therefore represents a new target to selectively inhibit protein translation in tumor cells.

## INTRODUCTION

hnRNP A18 is a RNA-binding protein that was first identified as a UV-inducible transcript in CHO cells more than two decades ago [1]. Since then, the hnRNP A18 protein has been characterized in human cells [2] and in mouse following exposure to mild cold shock. hnRNP A18 is thus also known as CIRP for Cold Inducible RNA Binding Protein [3]. Three hnRNP A18/CIRP mRNA transcripts, differing mainly in the size of their 5′UTRs, have been described [4]. The one we refer to as hnRNP A18 has the shortest 5′UTR and is expressed at 37°C. The two other transcripts are expressed at 32°C, harbor larger 5′UTRs and have shown internal ribosome entry segment (IRES)-like activity. hnRNP A18 is predominantly a nuclear protein but translocates to the cytoplasm in response to cellular stresses such as UV or hypoxia [5] [6]. It is now becoming apparent that hnRNP A18 up-regulation is associated with a large number of solid tumors. In fact, immunohistochemistry staining of a variety of tumors from 193 patients indicate that hnRNP A18 is up-regulated in about 30% of human tumors as compared to normal tissue from the same patients [7]. However, correlation with tumor grades or its potential tumor promoting activity in *in vivo* models has not been investigated so far.

Although a predominantly nuclear protein, hnRNP A18 has been located in the cytosol of several tumor cells [7]. This observation is consistent with the fact that most solid tumors develop hypoxic regions, mainly in the central core of the tumor, and that hnRNP A18 translocates to the cytosol in response to hypoxia [8] [9] [6]. Our earlier studies have revealed that hnRNP A18 translocation to the cytosol is mediated, in part, by the hypoxia inducible GSK-3β kinase and CK2 [10] [6]. GSK-3β also increases hnRNP A18 RNA binding activity and both, hnRNP A18 RNA binding domain and the RGG domain are required for maximal hnRNP A18 RNA binding activity [10]. In the cytosol, hnRNP A18 binds to a specific 51 nucleotide RNA signature motif present in the 3′UTR of targeted transcripts and increases their translation by interacting with eukaryotic initiation factor 4G (eIF4G), a component of the general translation cap-binding complex eIF4F, on polysomes [11] [10] [6]. Here, we investigated whether hnRNP A18 expression levels correlate with tumor grade and evaluated whether its regulatory role on protein translation under cellular stress could affect tumor growth and promotion. Our data indicate that hnRNP A18 stabilizes its targeted transcripts and increases *de novo* protein synthesis under hypoxic conditions. Moreover, hnRNP A18 is over expressed in 40 to 60% of human melanoma, prostate, breast and colon cancer tissue as compared to normal adjacent tissue and down regulation of hnRNP A18 decreases proliferation, invasion and migration in addition to significantly reducing tumor growth in two mouse xenograft models. To our knowledge, this is the first demonstration that hnRNP A18 can promote tumor growth in *in vivo* models. hnRNP A18 thus represents a new target to selectively inhibit protein translation in cancer cells and prevent human tumor growth.

## RESULTS

### hnRNP A18 up-regulation confers growth advantages to melanoma cells under hypoxic conditions

**Figure 1:**
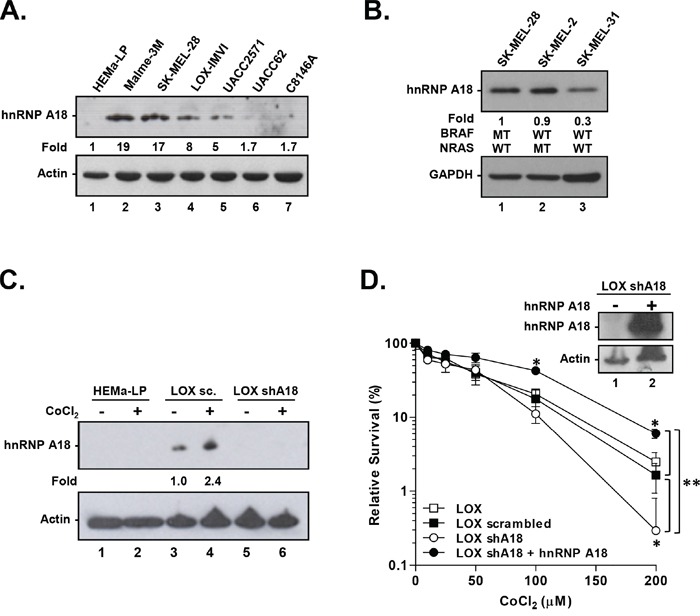
hnRNP A18 promotes tumor growth under hypoxic conditions Western blot analysis. **A.** Normal human melanocytes (HEMa-LP) and six melanoma cell lines were analyzed for hnRNP A18 levels. Fold induction was measured by densitometry and normalized to Actin. **B.** Melanoma cell lines harboring different BRAF/NRAS genotypes. The blots were hybridized with the indicated antibody. Fold induction was calculated by densitometry and normalized to GAPDH. **C.** Western Blot. Normal human melanocytes HEMa-LP or LOX-IMVI melanoma cells stably transfected with scrambled (LOX sc) or shhnRNP A18 RNA (LOX shA18) were exposed (+) or not to CoCl_2_ (100 μM) for 4 h. The blots were hybridized with the indicated antibody. Fold induction was calculated by densitometry and normalized to Actin. **D.** Relative survival assay performed on LOX-IMVI (open squares), LOX-IMVI stably transfected with scrambled RNA (closed squares), or shhnRNP A18 (open circles) or hnRNP A18 back in LOX-IMVI stably transfected with shhnRNP A18 (closed circles). Cells were exposed to the indicated concentration of CoCl_2_ and colonies were counted 10 days later. Relative survival is expressed as a percentage of colonies obtained with untreated cells. Inset (D) Western blot analysis. LOX-IMVI cells stably transfected with hnRNP A18 shRNA were re-transfected (+) with hnRNP A18 and analyzed by Western blots with the indicated antibody. * *p*<0.05, **p<0.005.

Given that hnRNP A18 was originally cloned on the basis of UV induction [1] and that it can confer resistance to UV-induced cellular death [5], we wanted to investigate whether hnRNP A18 could also be involved in the UV-induced skin cancer melanoma. The levels of hnRNP A18 protein were thus analyzed in six different melanoma cell lines and compared to normal human melanocytes (HEMa-LP). The data shown in Figure 1A indicate that hnRNP A18 is not detected in normal melanocytes but is over expressed in four of the six cell lines studied as compared to normal melanocytes. hnRNP A18 expression levels does not seem to be dependent on a BRAF mutant genotype since all the melanoma cell lines shown in Figure 1A harbor the BRAF mut/NRAS wild-type genotype and expressed different levels of hnRNP A18. Nonetheless, in order to verify whether hnRNP A18 levels are associated with BRAF or NRAS mutations we performed Western blot analysis with SK-MEL cell lines harboring different BRAF and NRAS genotypes. Data shown in Figure 1B indicate that hnRNP A18 is also expressed in melanoma cells harboring the BRAF wild-type genotype regardless of NRAS status (SK-MEL-2: BRAF wild-type, NRAS mutant; SK-MEL-31: BRAF wild-type, NRAS wild-type). It thus appears that hnRNP A18 levels are not dependent on BRAF genotype. Because hnRNP A18 can translocate from the nucleus to the cytosol in response to the hypoxia mimetic agent CoCl_2_ [6] and that fifty to sixty percent of locally advanced solid tumors, including melanoma, develop hypoxic areas, we next investigated whether hypoxic conditions could affect hnRNP A18 levels. Our data (Figure 1C) indicate that hnRNP A18 levels increase following CoCl_2_ exposure in melanoma but not in normal melanocytes (lanes 4 and 2). Down regulation of hnRNP A18 with shRNA abolished hnRNP A18 induction by CoCl_2_ (lanes 5 and 6). We then measured the effect of hnRNP A18 expression on the cells' capacity to survive CoCl_2_ exposure. The data shown in Figure 1D indicate that down regulation of hnRNP A18 significantly increased LOX-IMVI sensitivity to CoCl_2_, while re-expressing hnRNP A18 in cells that were stably transfected with shhnRNP A18 (inset Figure 1D) rescued CoCl_2_ resistance at every dose tested (Figure 1D). These data show for the first time that hnRNP A18 is over expressed in melanoma cells and that it could provide growth advantages to tumors.

### hnRNP A18 confers a growth advantage to cancer cells *in vivo*

**Figure 2:**
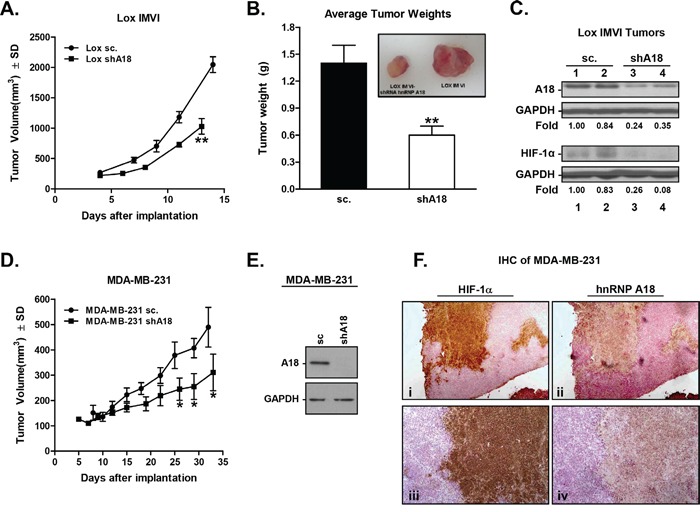
hnRNP A18 promotes human tumor growth *in vivo* Mouse Xenograft models: **A.** LOX-IMVI melanoma xenografts. LOX-IMVI cells stably transfected with either scrambled RNA or shhnRNP A18 were injected s.c. in the flanks of athymic mice (nu/nu). Tumor volumes were measured by caliper at the indicated intervals. **B.** Tumors were excised and weighed 12-15 days later. Average tumor weight from seven animals. Black bar; tumor from LOX-IMVI stably transfected with scrambled RNA (sc), white bar; tumor from LOX-IMVI cells stably transfected with shhnRNP A18. Inset: Left-hand side tumor formed with LOX-IMVI cells stably transfected with shhnRNP A18 injected on the left flank. Right-hand side, tumor formed in the same animal injected on the right-hand side with LOX-IMVI cells. **C.** Western blots analysis from tissue excised from tumors formed *in vivo*. Each lane indicates analysis performed on tissue extracted from same tumor with the indicated antibody. Fold induction was calculated by densitometry and normalized to GAPDH. **D.** Same as in A) except that breast MDA-MB-231 cells were used. **E.** Western blots performed on the indicated MDA-MB-231 cells before injections into the mice. **F.** Immunohistochemistry performed on a tumor formed in a mouse xenograft injected with MDA-MB-231 cells stably transfected with scrambled RNA. Two consecutive microtome sections from the same tumor were stained with either HIF-1α (i, iii) or hnRNP A18 (ii, iv) and counter stained with Mayer's Hematoxylin and mounted on coverslips. Magnification; 4X (i, ii) and 10 X (iii, iv). *p<0.05, **p<0.005

To verify the functional significance of these findings, we evaluated the effects of hnRNP A18 down regulation on tumor growth *in vivo*. Melanoma LOX-IMVI cells stably transfected with shhnRNP A18 were injected in the left flanks of athymic mice (nu/nu) while LOX-IMVI cells stably transfected with a scrambled shRNA were injected in the right flanks of the same animals. The data shown in Figure 2A-2B indicate that down regulation of hnRNP A18 significantly reduces melanoma tumor growth by about 50% and tumor weight by about 60% as compared to control (scrambled) tumors. Tumor progression (Figure 2A) indicates that the difference between control (scrambled) and shhnRNP A18 tumors is more pronounced after the first week of tumor growth. Because hnRNP A18 is translocated to the cytosol in response to hypoxic conditions [6] and can regulate expression of stress-responsive transcripts, we next evaluated whether hnRNP A18 could affect the expression of HIF-1α, a key regulator of the cellular response to hypoxia. Data from tissue extracted from four tumors grown *in vivo* from cells expressing reduced (Figure 2C, shA18, lanes 3, 4) or endogenous (sc, lanes 1, 2) hnRNP A18 levels indicate that down regulation of hnRNP A18 prevents upregulation of HIF-1α in the tumors. To determine whether the effect of hnRNP A18 on tumor growth was cell type specific, we also performed a tumor progression experiment with breast cancer cells. The data shown in Figure 2D indicate that down regulation of hnRNP A18 significantly reduced breast cancer tumor growth as compared to tumors formed with MDA-MB-231 cells stably transfected with scrambled RNA (Figure 2E). Moreover, immunohistochemistry staining of the breast cancer tumors formed *in vivo* indicate that hnRNP A18 accumulates in the hypoxic region of the tumor as defined by HIF-1α staining (Figure 2F). These data thus suggest that over expression of hnRNP A18 in tumor cells promotes tumor growth.

### hnRNP A18 promotes proliferation, invasion and migration

**Figure 3:**
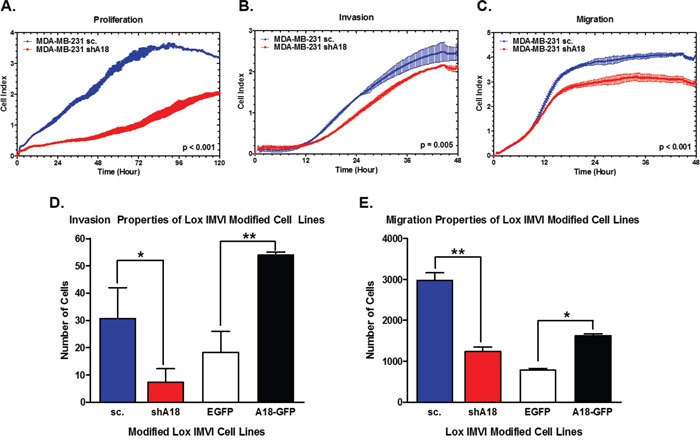
hnRNP A18 increases proliferation, invasion and migration **A.** Proliferation, **B.** Invasion and **C.** Migration assays performed on breast MDA-MB-231 cells stably transfected with shhnRNP A18 (red curves) or scrambled RNA (blue curves). Assays were performed on xCELLigence as described in the text. For all assays, events were measured by cell-sensor impedance, expressed as arbitrary unit Cell Index (CI), defined as (RnRb)/15, where Rn is the cell-electrode impedance of a cell-containing well and Rb is background impedance in wells containing only media. P values are as indicated. **D.** Invasion assay performed on melanoma LOX-IMVI cells stably transfected with shhnRNP A18 (red bars), scrambled RNA (blue bars), GFP-hnRNP A18 (black bars) or EGFP (white bars). Assays were performed in Corning Matrigel Invasion chambers as described in the text. **E.** Migration assays performed as described in D) except that the assays were performed in Transwell Boyden chambers. *p< 0.05, **p< 0.005

To determine if hnRNP A18 over expression in human tumors could affect proliferation, invasion and/or migration, we monitored these parameters in real-time with an xCELLigence RTCA MP instrument by measuring cell sensor impedance on cells expressing endogenous or reduced hnRNP A18 levels (shA18). The data shown in Figure 3A-3C indicate that down regulation of hnRNP A18 significantly reduced breast cancer cells' proliferation, invasion and migration. Similar data were obtained by conventional invasion and migration assays performed with melanoma cells. The data shown in Figure 3D-3E indicate that down regulation of hnRNP A18 significantly reduces melanoma cells' invasion and migration properties while over expression of hnRNP A18 significantly increases these parameters.

### hnRNP A18-mediated growth advantages is mediated in part through HIF-1α regulation

**Figure 4:**
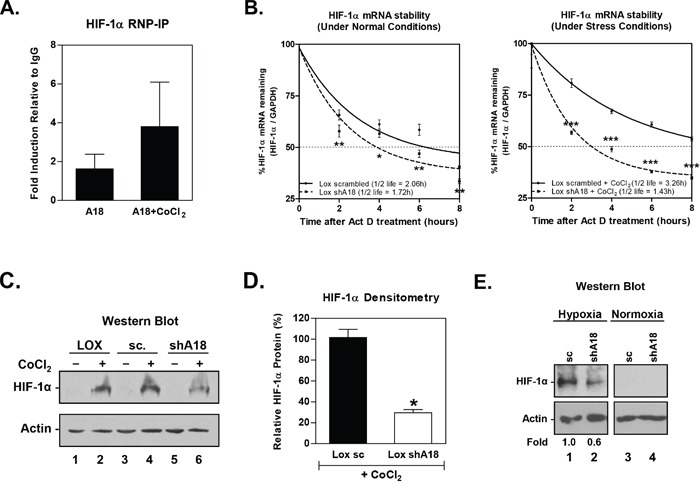
hnRNP A18 increases HIF-1α translation **A.** Ribonucleoprotein Immunoprecipitation (RNP-IP) on polysomes extracted from cells exposed or not to CoCl_2_. IP was followed by RT-PCR (26 cycles) to detect endogenous HIF-1α transcript. GAPDH was amplified from the same fractions to confirm that equal amounts of mRNA were present in each immunoprecipitated sample. Densitometry results of three independent experiments were normalized to GAPDH and to the amount of HIF-1α mRNA immunoprecipitated with IgG antibody. **B.** mRNA stability assay. LOX-IMVI cells stably transfected with either scrambled RNA (solid line or shhnRNP A18 (dashed line) were treated (right panel) or not (left panel) with CoCl_2_ for 4 hours, then Actinomycin D (10 μg/ml) was added. RNA was collected at the indicated time points, reverse transcribed and analyzed by Real-Time PCR. Data were normalized to GAPDH mRNA and expressed as a percentage of the zero time point of each respective sample group on nonlinear regression to determine half-life. **C.** Western blot analysis performed on LOX-IMVI cells (LOX) stably transfected with either scrambled shRNA (sc) or hnRNP A18 shRNA (shA18) were exposed (+) or not (−) to CoCl_2_ (100 μM, 4h). Positions of HIF-1α and Actin are indicated. **D.** Relative levels of HIF-1α measured by densitometry from Western blots. Levels of HIF-1α expression are expressed as a percentage of the LOX-IMVI sc cells exposed to CoCl_2_. **E.** Same as C) except that the cells were exposed to 0.5% O_2_ (hypoxia) or 20% O_2_ (normoxia) for 24 h. *p<0.05, **p<0.005, ***p<0.0005

To determine whether hnRNP A18 could modulate HIF-1α levels and consequently cells' sensitivity to the hypoxic environment, we first evaluated whether hnRNP A18 could bind to the HIF-1α transcript. We performed RNA immunoprecipitation on polysomes extracted under conditions that preserved the association of RNA-binding proteins with target mRNA, essentially as described before [10] [12]. hnRNP A18-bound mRNAs were then eluted and used for RT-PCR to amplify the products. The data shown in Figure 4A indicate that hnRNP A18 does bind to HIF-1α mRNA in the absence and presence of CoCl_2_. However, although the amount of HIF-1α mRNA pulled down by hnRNP A18 antibody in the presence of CoCl_2_ (A18 + CoCl_2_) is apparently higher than in the absence of CoCl_2_, the difference is not statistically significant. Nonetheless, hnRNP A18 could potentially affect HIF-1α transcript stability. To verify this possibility, we performed mRNA stability experiments in the presence of Actinomycin D and reduced amounts of hnRNP A18. Our data (Figure 4B) indicate that in absence of CoCl_2_, HIF-1α mRNA half-life is 2.06h. Down regulation of hnRNP A18 reduces this half-life to 1.72h for a 0.34h difference. In the presence of CoCl_2_, HIF-1α half-life increases to 3.26h and down regulation of hnRNP A18 reduces it to 1.43h for a difference of 1.83h. Therefore, our data indicate that the binding of hnRNP A18 HIF-1α mRNA in the presence of CoCl_2_ (Figure 4A) significantly increases HIF-1α mRNA half-life by almost 2h. We next determined if the interaction of hnRNP A18 with HIF-1α mRNA is affecting HIF-1α protein levels. The data shown in Figure 4C indicate that HIF-1α is over expressed in response to CoCl_2_ (lanes 2 and 4) but down regulation of hnRNP A18 significantly reduces HIF-1α protein levels in response to CoCl_2_ (lane 6 and Figure 4D). Similar data were also obtained when cells were grown under hypoxia (0.5% O_2_) where down regulation of hnRNP A18 resulted in reduced HIF-1α protein expression levels (Figure 4E). These data are in good agreement with Figure 2F showing expression of hnRNP A18 in the hypoxic area of human tumors where HIF-1α is expressed and likely to be regulated by hnRNP A18.

### hnRNP A18 affects angiogenesis factors

**Figure 5:**
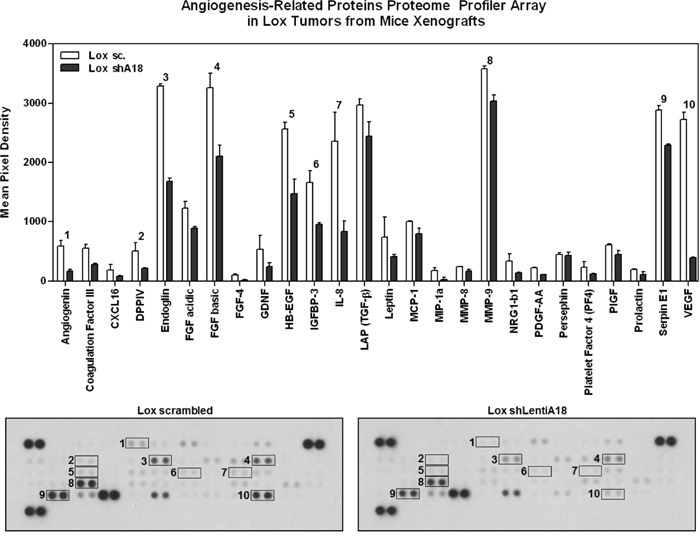
Angiogenesis Proteome Profiler Array Lox tumors expressing endogenous (sc; white bars) or reduced (sh; black bars) hnRNP A18 levels were excised from mouse xenografts, proteins extracted and analyzed as described in the text. Expression levels of angiogenesis proteins were measured by densitometry and expressed as mean pixel density after subtracting background.

One of the main contributions of HIF-1α to tumor promotion is the upregulation of angiogenic growth factors that provide oxygen to the hypoxic tumors [13]. To determine whether hnRNP A18 could affect HIF-1α functions in growing tumors *in vivo* we evaluated the angiogenic proteomes of melanoma tumors expressing endogenous or reduced hnRNP A18 levels. Of the fifty-five angiogenic factors evaluated, about half including angiogenin, endoglin and most specifically VEGF (Figure 5, box 10), a prime HIF-1α regulated growth factor, were down regulated in the shhnRNP A18 tumor as compared to control. These data are in good agreement with the *in vivo* data (Figure 2) indicating that down regulation of hnRNP A18 prevent tumor growth. To further confirm that HIF-1α could contribute to hnRNP A18 growth promoting activity, we down regulated HIF-1α in melanoma cells and measured cell viability in the presence of CoCl_2_. Our data (Supplemental Figure 1) indicate that indeed down regulation of HIF-1α significantly reduces cell viability in the presence of CoCl_2_.

### hnRNP A18 RNA binding signature motif in cancer promoting genes

**Figure 6:**
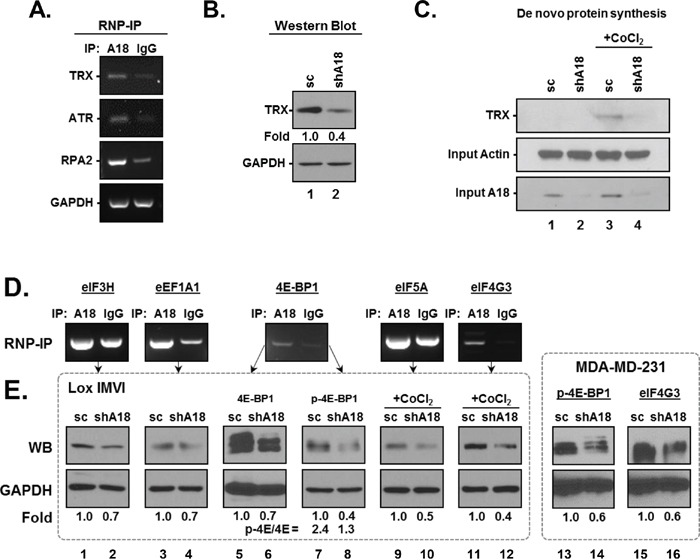
hnRNP A18 targeted transcripts **A, D.** RNA bound to Ribonucleoprotein was immunoprecipitated (RNP-IP) from LOX-IMVI cells with the indicated antibody. GAPDH was amplified from the same fractions to confirm that equal amounts of mRNA were present in each immunoprecipitated sample. **B.** Western blot analysis performed in LOX-IMVI cells stably transfected with scrambled (sc) or shhnRNP A18 RNA (shA18) with the indicated antibodies. Fold induction was calculated by densitometry and normalized to GAPDH. **C.** *De novo* protein synthesis. LOX-IMVI cells stably transfected with scrambled (lanes 1, 3) or shhnRNP A18 RNA (lanes 2, 4) were exposed (lanes 3, 4) or not (lanes 1, 2) to CoCl_2_ in methionine-free media and labeled with Click-iT® Metabolic Labeling Reagents as described in the text. TRX antibody was incubated with the newly biotinylated protein and detected by chemiluminescent with anti-Streptavidin-HRP antibody. Actin was used as loading control. **E.** Western blot analysis performed as in B) in LOX-IMVI cells (lanes 1-12) and MDA-MB-231 cells (lanes 13-16) with the indicated antibodies. Fold induction was calculated by densitometry and normalized to GAPDH. Ratio of p-4E-BP1 over 4E-BP1 was calculated by densitometry (lanes 5-8) normalized to GAPDH.

The hnRNP A18 RNA binding signature motif has been identified and validated in a number of transcripts associated with tumor progression [10] [6]. As with most key regulatory proteins mediating survival benefits, we expect that the transcripts targeted by hnRNP A18 will mediate different cellular functions that will result in an overall growth advantage to the tumor when coordinately upregulated. A search of the UniGene database revealed that indeed the hnRNP A18 RNA signature motif could be located in the 3′UTRs of transcripts associated with proliferation, survival and invasion (Supplemental Figure 2). ATR, RPA2 and TRX have been validated before as hnRNP A18 targets in colon cancer cells [5] [10] [6] and are used here as positive controls for hnRNP A18 binding to targeted transcripts in melanoma cells (Figure 6A). The data shown in Figure 6A indicate that TRX transcript is pulled down by hnRNP A18 antibody but not the nonspecific IgG antibody in melanoma cells. Western blot analyses indicate that down regulation of hnRNP A18 reduced the basal protein levels of TRX in melanoma cells (Figure 6B). Because hnRNP A18 is associated with low molecular weight polysomes [10], we next wanted to evaluate the effect of hnRNP A18 on newly synthesized proteins. The data shown in Figure 6C indicate that down regulation of hnRNP A18 prevents the accumulation of newly synthesized TRX protein in the presence of the hypoxia mimetic agent CoCl_2_ (lane 3-4). These data thus support the notion that hnRNP A18 increases mRNA stability (Figure 4B) and translation of its targeted transcripts under cellular stress conditions.

Our search of the UniGene database also identified several members of the general translational machinery as potential hnRNP A18 targets (Supplemental Figure 2). RNP-IP performed with hnRNP A18 antibody validated this prediction and indicated that indeed hnRNP A18 could efficiently bind to eIF3H, eEF1A1, eIF4E-BP1, eIF5A and eIF4G3 (Figure 6D). Western blot analyses indicate that down regulation of hnRNP A18 reduced the basal protein levels of its targeted transcripts in melanoma and breast cancer cells (Figure 6E). Of particular interest is the effect of hnRNP A18 down regulation on eIF4E-BP1. When phosphorylated, eIF4E-BP1 relieves the translational repression imposed on eIF4E and allows translation to proceed. By regulating eIF4E-BP1 levels, hnRNP A18 also affects the levels of phosphorylated eIF4E-BP1 (p-eIF4E-BP1, Figure 6E, lanes 7-8) and could affect translation. In fact, the ratio of p-eIF4E-BP1 over total eIF4E-BP1 decreases by almost 50% in cells expressing reduced hnRNP A18 levels (Figure 6E, lanes 5-8). In order to determine if the regulatory function of hnRNP A18 under cellular stress was specific to TRX and HIF-1α we also evaluated the protein levels of eIF4G3 and eIF5A in the presence of CoCl_2_. Our data indicate that down regulation of hnRNP A18 reduces the protein levels of these two transcripts in the presence of CoCl_2_ (Figure 6E, lanes 9-12).

### hnRNP A18 is over expressed in human tumors including melanomas

**Figure 7:**
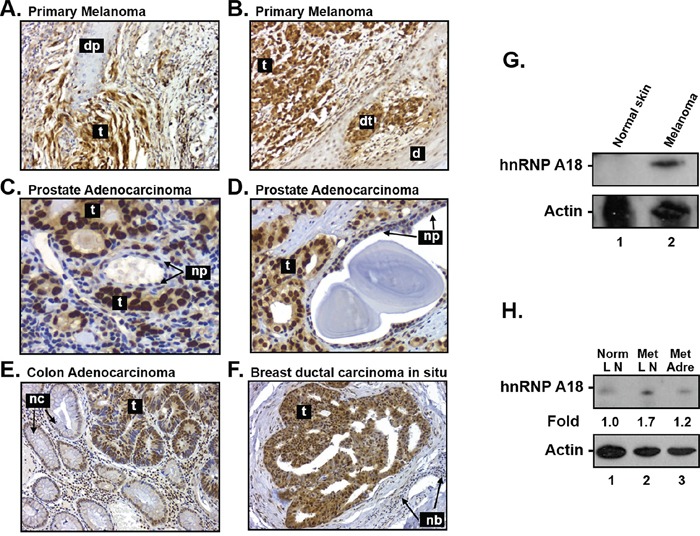
hnRNP A18 expression in human tumors Tissue microarrays (TMA). A-B. Immunohistochemistry of primary melanoma with built-in adjacent normal skin. **A.** Stained tumor cells (t) near negative dermal papillae (dp). **B.** Tumor cells (t), including tumor cells in the derma (dt) were strongly positive for hnRNP A18 while adjacent normal skin (d) showed no or very weak staining. **C-D.** Prostate adenocarcinoma shows strong nuclear staining in the tumor cells (t) while adjacent normal prostate (np) showed no or very weak staining. **E.** Colon adenocarcinoma with built-in adjacent normal colon shows strong nuclear hnRNP A18 staining in the tumor cells (t) but weak staining in the adjacent normal colon (nc) where well defined lobular pattern of colonic crypts divided by smooth muscle bundles can be observed. **F.** A ductal carcinoma *in situ* shows strong hnRNP A18 nuclear staining in tumor cells (t) while adjacent normal breast (nb) showed no or very weak staining. G-H) Western blots from human tissues. **G.** Proteins were extracted from normal skin or melanoma skin tumors and analyzed by Western blots with the indicated antibodies. **H.** Same as G) except that the proteins were extracted from normal lymph nodes (Norm LN) or metastatic melanoma tumors to the lymph nodes (Met LN) or the adrenals (Met Adre). Fold induction was calculated by densitometry and normalized to Actin.

hnRNP A18 (CIRP) levels are elevated in several human tumors, but to our knowledge, hnRNP A18 levels in primary melanoma and correlation to tumor grades have not yet been investigated [7]. In order to evaluate the potential clinical significance of hnRNP A18 upregulation, we performed immunohistochemistry on tissue microarrays (TMA) from 295 human samples including 70 primary and metastatic melanoma samples (Table 1). Among primary melanomas, 42% showed moderate staining and 42% showed strong/very strong staining, while adjacent normal skin showed no or very weak staining. Representative staining patterns are shown in Figure 7 where stained primary melanoma tumor cells near negative dermal papilla (Figure 7A) and tumor cells in the derma (Figure 7B) were strongly positive for hnRNP A18, while adjacent normal skin showed no or weak staining. In metastatic melanoma cases, 58% showed moderate to strong staining while 42% were negative. Comparable patterns of expression were also observed in colon, prostate, and breast cancer TMA samples (Figure 7A–7F, Table 1) where higher hnRNP A18 expression was observed in the cancer cells as compared to normal adjacent tissue. Although no apparent correlation with tumor grades was observed in colon cancer, stronger hnRNP A18 staining was observed in more advanced prostate and breast cancers as compared to non-cancer prostate (prostate hyperplasia) and noninvasive breast cancer tissues (ductal carcinoma *in situ*) (Table 1). While hnRNP A18 is predominantly a nuclear protein, staining was exclusively cytoplasmic in 20% of malignant melanomas and always nuclear in normal cells. This is consistent with the capacity of hnRNP A18 to translocate to the cytosol in response to cellular stress such as UV radiation and hypoxia. These immunohistochemistry data are in good agreement with the levels of hnRNP A18 protein as measured by Western blots in human melanoma tumor tissues (Figure 7G–7H). hnRNP A18 protein levels were found to be elevated in primary melanoma as compared to normal skin tissue and slightly more elevated in melanoma which metastasized to the lymph nodes as compared to protein extracted from normal lymph nodes (Figure 7G–7H).

**Table 1:** hnRNP A18 protein expression in human cancer as evaluated by immunohistochemistry on Tissue Micro arrays (TMAs)

Cancer Type	Staining scores				% Location				Total No of patients
	% Negative	% Weak	% Moderate	% Strong/Very strong	N	N/C	C/N	C	
**Melanoma**									
Normal skin	43	14	14	29	100				7
Primary	4	12	42	42	13	26	39	22	46
Metastatic Lymph Node	42		16	42	57	43			24
**Prostate**									
Hyperplasia	0	0	80	20	80	20			10
Adenocarcinoma	0	0	36	64	64	36			28
Metastatic rectum	0	100	0	0	100				2
**Breast**									
Ductal carcinoma *In situ*	0	17	50	33	50	50			6
Invasive ductal carcinoma	12	15	33	40	24	75			77
Invasive lobular carcinoma	0	0	50	50	43	57			8
**Colon**									
Normal colon	50	50	0	0	100				2
Malignant Grade I	25	25	25	25	50	50			4
Malignant Grade I-II	22	15	26	37	48	22		3	27
Malignant Grade II	21	18	34	27	87	13			33
Malignant Grade II-III	40	10	10	40	10	30	10		10
Malignant Grade III	27	36	28	9	100				11
Total									295

Blank spaces indicate that no staining was detected.

## DISCUSSION

Targeting protein translation for cancer therapy is an attractive strategy to limit cancer cells proliferation by depriving them of essential nutriments. Inhibitors of the mTOR pathway have in fact shown clear benefits in some cancers such as Mantle Cell Lymphomas, Renal Cell Carcinoma and Tuberous Sclerosis Complex-related tumors but have limited efficacy as single agents in most other cancers [14]. Feedback mechanisms that can compensate for this general protein translation pathway and the specificity of the targeted substrates are among the main limitations for these drugs' efficacy. Our previous reports [5] [10] [6] and the data presented here indicate that hnRNP A18 regulates tumor growth by preferentially targeting translation of specific mRNA transcripts harboring its RNA signature motif in response to cellular stress. hnRNP A18 targets transcripts such as HIF-1α, RPA2, TRX, and ATR that can confer growth advantages under cellular stress like UV radiation [5] [10] and hypoxia (Figure 2, 4). In addition, the data presented here indicate that hnRNP A18 also targets a number of transcripts associated with the general translational machinery. hnRNP A18 thus appears to promote tumor growth by coordinating the translation of functionally related transcripts associated with proliferation, survival and invasion. In the context of RNA-binding proteins, this phenomenon is known as the RNA regulon or operon where mRNAs that encode functionally related proteins are coordinately regulated as post-transcriptional RNA operons or regulons, through a ribonucleoprotein-driven mechanism [15]. For example, the coordinate upregulation of HIF-1α and TRX is likely to mediate the increase survival effects observed *in vivo* while upregulation of protein translation factors will support the demand of actively proliferating cells. hnRNP A18's effect on tumor promotion thus appears to be the result of its overall impact on its targeted transcripts.

**Figure 8:**
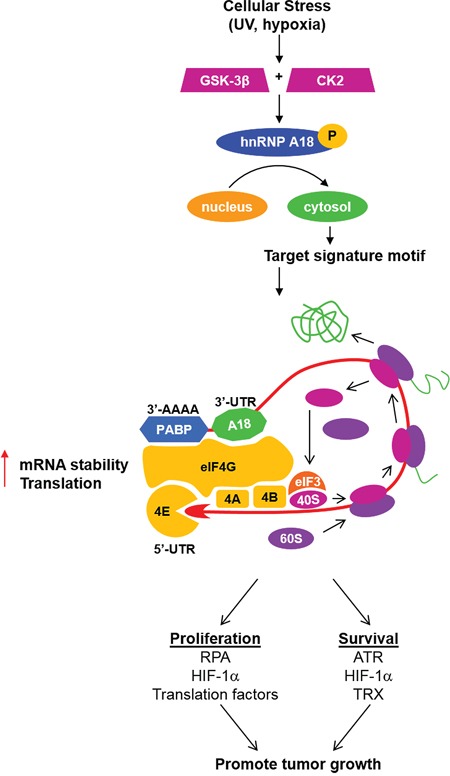
Based on our published observations and the data presented here we propose the following mechanism for hnRNP A18 tumor promoting activity In response to cellular stress such as UV radiation and hypoxia, the predominantly nuclear hnRNP A18 is phosphorylated by GSK-3β and CK2 and translocates to the cytosol [5] [6]. In the cytosol, hnRNP A18 recognizes a specific signature motif in the 3′UTR of the targeted transcripts and increases their translation by associating with eIF4G on polysomes [10] [6]. In response to cellular stress, hnRNP A18 up-regulates the translation of transcripts conferring tumor growth advantages such as HIF-1α, RPA, TRX, ATR and components of the general translational machinery. PABP: PolyA Binding Protein. The arrows on the right panel indicate the direction of ribosome scanning and translating.

Although hnRNP A18 binds to the 3′UTR of its targeted transcripts [5] [10] [6], our data suggest that hnRNP A18 regulates translation at the initiation phase. This is supported by the fact that hnRNP A18 associates with low molecular weight polysomes [10] and interacts with eIF4G [10]. Based on our published data and the data presented here, we propose the model depicted in Figure 8. In response to cellular stress, the predominantly nuclear hnRNP A18 is phosphorylated and translocated to the cytosol. In the cytosol, hnRNP A18 binds to its targeted transcripts, stabilizes the transcripts and increase their translation (Figures 4, 6). Increased mRNAs stability is however probably not sufficient to increase translation since general protein translation is inhibited under hypoxic condition [16]. Our previous data have indicated that hnRNP A18 can bridge its targeted transcripts to the general translational machinery through interaction with eIF4G [10]. In addition, hnRNP A18 can modify the ratio of eIF4E-BP1 and phosphorylated eIF4E-BP1 to facilitate translation (Figure 6E). Nonetheless, we cannot completely rule out a possible involvement in protein elongation since hnRNP A18 also binds to elongation factor eEF1A1 mRNA (Figure 6). hnRNP A18 thus provides a new mechanism to counteract, at least in part, the inhibitory effect of hypoxia on general protein translation by targeting survival factors harboring its RNA signature motif. However, it seems unlikely that hnRNP A18 could affect protein translation in normal cells since hnRNP A18 is primarily nuclear under normal conditions [5] [6] and it is expressed at very low levels in these cells (Figure 1C, Figure 7A-7F). Moreover, hnRNP A18 is not upregulated by hypoxic conditions in normal cells (Figure 1C). The mechanisms that may lead to hnRNP A18 upregulation in response to hypoxia in normal cells are thus likely to be different than in cancer cells. As with other stress responsive proteins we expect that oncogenic pathways characteristic of cancer cells will contribute to hnRNP A18 upregulation in response to hypoxia. Nonetheless, it is also possible that spatio-temporal controls could be involved [17]. Most importantly, this differential expression pattern could potentially represent a therapeutic advantage where targeting hnRNP A18 would primarily affect cancer cells progression while sparing normal cells functions.

The role that cellular stress plays in hnRNP A18 up-regulation is likely to be similar in most solid tumors but is expected to be particularly important for melanoma. In fact, levels of HIF-1α correlate with melanoma growth [18], and inhibition of ATR has been shown to increase apoptosis in UV-exposed primary human keratinocytes [19]. Here, we show that hnRNP A18 is expressed in the hypoxic area of tumors (Figure 2F) and that it can regulate the expression of HIF-1α by stabilizing its transcript (Figure 4B). It thus seems conceivable that the hypoxic regions that develop in solid tumors contribute to hnRNP A18 up-regulation in the tumor and consequently up-regulation of its targeted transcripts such as HIF-1α. In fact, proteome analysis of angiogenic factors indicate that key HIF-1α regulated angiogenic factors such as VEGF are down regulated in tumors expressing reduced hnRNP A18 levels (Figure 5). However, it remains to be determined whether these factors could also be directly regulated by hnRNP A18. The data generated so far indicate that hnRNP A18 stabilizes its targeted transcripts and increases their translation, but as it is the case for other stress responsive RNA-binding proteins such as HuR it remains possible that some potential anti-proliferative targets could be destabilized and down regulated for an overall growth advantage effect [20].

hnRNP A18 could also promote tumor growth by regulating the expression of key regulators of the cellular oxidation-reduction homeostasis. We have previously shown that hnRNP A18 binds to its RNA signature motif in the 3′UTR of Thioredoxin (TRX) and regulates TRX protein levels [10], (Figure 6). TRX increases the expression and activity of HIF-1α which is associated with poorer prognosis and metastatic potential in certain cancers [21] [22]. hnRNP A18 could thus regulate HIF-1α expression indirectly through TRX and directly through binding to its mRNA transcript (Figure 4A). A positive feedback loop could also develop between hnRNP A18 and HIF-1α since hnRNP A18 binds to and stabilizes HIF-1α transcript (Figure 4A) and several HIF-1α consensus binding sites are located in the sequence preceding hnRNP A18 mRNA [23]. However, hnRNP A18 up-regulation in response to hypoxia is independent of HIF-1α [9]. Nonetheless, hypoxic conditions could provide an amplification environment whereby hnRNP A18 could up-regulate HIF-1α and HIF-1α could in turn contribute to sustaining hnRNP A18 levels during prolonged hypoxia. Our *in vivo* data (Figure 2) support this notion and suggest that hnRNP A18 confers growth advantages to established tumors rather than being part of the tumor initiating phenotype. This concept is further supported by the low basal and un-inducible levels of hnRNP A18 in normal melanocytes (Figure 1) and the greater difference between the tumor volumes of scrambled and shhnRNP A18 tumors as time progresses (Figure 2). It thus seems conceivable that once a metastatic tumor is established and hypoxic areas have developed, hnRNP A18 could become a contributing factor in sustaining cancer progression.

Our study mainly focused on the intracellular role of hnRNP A18 in cancer progression, but our TMA analysis indicated that hnRNP A18 is also present in the stroma of all cancer tissues we evaluated (Figure 7A–7F). This suggests that under certain conditions hnRNP A18 may be secreted and could potentially contribute to maintain cancer progression and/or propagation. In fact, a recent study indicated that hnRNP A18 is secreted into the blood in response to hemorrhagic shock and sepsis and triggers an inflammatory response [24]. hnRNP A18 is thus likely to play a similar role in cancer cells by promoting an inflammatory response in the stroma. Actually, a recent study indicated that hnRNP A18/CIRP contributed to intestinal inflammation and colitis-associated cancer [25]. It remains to be determined whether this inflammatory response is contributing to the promotion of cellular invasion and migration (Figure 3). One possible mechanism by which hnRNP A18 could contribute to cellular migration and proliferation is through regulation of TRX and HIF-1α which both have been associated with these tumor promoting events.

Taken together, our data suggest that hnRNP A18 can coordinate the regulation of a variety of transcripts to support the demands of proliferating cells (protein translation), increase survival (ATR, TRX, HIF-1α) and proliferation (RPA, HIF-1α) under cellular stress. Rational targeting of protein translation through hnRNP A18 inhibition could thus provide a new mechanism to deprive cancer, but not normal cells, of nutriments essential to sustain growth and proliferation. Identification of small molecule inhibitors that could target hnRNP A18 would therefore be desirable to further explore these possibilities.

## MATERIALS AND METHODS

### Cell lines and treatments

Human malignant melanoma cell lines were obtained and grown as described previously [6]. The normal Human Epidermal Melanocytes, adult, HEMa-LP were purchased from Life Technology (Invitrogen) and maintained in Medium 254 supplemented with Human Melanocyte Growth Supplement-2 (HMGS-2). The human breast cancer cell line MDA-MB-231 was purchased from ATCC (Manassas, VA) and grown in DMEM (Dulbeco's Modified Eagle Medium) with high glucose supplemented with 10% Fetal Bovine Serum.

### Cells proliferation, invasion and migration

Real-time monitoring of proliferation, invasion, and migration were performed on the xCELLigence RTCA MP Instrument from ACEA Biosciences, Inc. (ACEA) according to manufacturer's instructions. For the proliferation assay, MDA-MB-231 scrambled and MDA-MB-231 shLentiA18 cells (5,000 cells/well) were plated in triplicates in 96X microplates (E-Plate) and monitored every 15 minutes for a total of 120 hours. In the invasion and migration experiments, MDA-MB-231 scrambled and MDA-MB-231 shLentiA18 cells (75,000 cells/well) were seeded without FBS into the upper chamber of a CIM-Plate 16. Complete growth medium supplemented with 10% FBS (or no FBS as negative control) was added in the lower chamber as a chemoattractant. CIM-Plate 16 was loaded into the xCELLigence RTCA MP Instrument for real-time monitoring at 15-minute intervals for 48 hours. Conventional migration assays were carried out in Transwell Boyden chambers (Corning costar) with polycarbonate filters (8 μm pores). LOX-IMVI (2.5×10^4^) cells stably expressing scrambled or shhnRNP A18, were added to the upper chamber without serum while the lower chamber contained growth media with 10% FBS. After incubation for 24h at 37°C, non-migrating cells were removed by sterile cotton swab. Cells that migrated to the lower surface were fixed in 100% methanol and stained with 0.1% (w/v) crystal violet solution. Images of cells from three different areas were taken and counted. Experiments were done twice in triplicates and data was presented as the average number of cells which migrated. Matrigel invasion assay was carried out essentially as described above (migration assay) except that Corning Matrigel Invasion chambers with polycarbonate filters (8 μm pores) were used.

### Cells transfection

LOX-IMVI cells were stably transfected (FuGene HD, Promega) with four different shRNA vectors of hnRNP A18 or a mixture of the four vectors (1 μg total, OriGene, Rockville, MD) or scrambled RNA and selected with Puromycin (0.5 μg/ml) for 2 weeks. Where indicated, stable LOX-IMVI cells transfected with shhnRNP A18 were re-transfected with hnRNP A18-GFP [5] and selected with Hygromycin (50 μg/ml) for two weeks. Parent LOX-IMVI cells were also stably transfected with hnRNP A18-GFP and selected with Hygromycin for two weeks. For animal studies, LOX-IMVI and MDA-MB-231 cells were stably transfected with scrambled or shhnRNP A18 lentivirus with Mission lentiviral packaging mix (Cat #SHP 001, Sigma Aldrich, St. Louis, MO) according to the manufacturer's instructions. Briefly HEK-293T cells were transfected with pGFP-C-shhnRNP A18 lenti (TL313897 A-D) or pGFP-CSh scrambled lenti (TR-30021) (OriGene, Rockville, MD) and packaging plasmids using FuGene 6 (Promega). Detection of GFP expression and Western blotting for hnRNP A18 were carried out to determine infection efficiency.

### Animal studies

LOX-IMVI and MDA-MB-231 cells stably transfected with either a vector expressing a scrambled shRNA sequence or a mixture of four plasmids expressing different hnRNP A18 shRNAs (LOX-IMVI) or shhnRNP A18-B (MDA-MB-231) were injected s.c. in the flanks of six to eight four-week-old female athymic mice (nu/nu). The mice received 3 × 10^6^ cells transfected with shhnRNP A18 in 100 μl plus matrigel in the left flank or 3 × 10^6^ LOX-IMVI stably transfected with scrambled shRNA on the right flank. Tumors were allowed to grow for 15 to 35 days, volumes measured by caliper at different intervals, then the mice were sacrificed and the tumors were excised and weighed.

### Clonogenic survival assays

Clonogenic survival assays were performed as described previously [5]. Briefly, 3 × 10^2^ cells were plated in 6-well plates and exposed to increasing concentrations of CoCl_2_ for 48 h. The cells were then replenished with fresh media and colonies were counted 7-10 days later. Treatments were performed in triplicate for each dose.

### Tissue microarrays

Immunohistochemistry was performed on sections of 295 primary human tumors with built-in adjacent tissues. The slides were prepared with an immunohistochemistry kit (Pantomics Mini IHC Kit) according to the manufacturer recommendations. Briefly, the slides were baked in an oven at 60°C for 60 min and deparaffinized with xylene for 5 min, twice. The sections were then rehydrated through a series of washes with decreasing ethanol concentration (100-70%) and soaked in water. Endogenous peroxide activity was inactivated and heat-induced antigen retrieval was performed. The slides were then heated for 5 min in a conventional pressure cooker at moderate power. After incubating the slides in pre-blocking solution, the slides were incubated with hnRNP A18/CIRP rabbit polyclonal antibody (Abcam) at 1/1000 and 1/2000 dilutions for 1h. Secondary antibody conjugated to horseradish peroxidase was then used and the slides were reacted with DAB substrate. The sections were counterstained with Mayer's Hematoxylin and mounted on coverslips. Rabbit antibody to cytokeratin and normal rabbit serum were used as positive and negative controls. Immunohistological staining of tissue microarrays for hnRNP A18 was scored blindly by a pathologist at Pantomics, Inc. (Richmond, CA) using the following criteria: “0” = negative; “0.5” = negative with some weak but suspicious staining; “1” = weak staining; “2” = moderate staining; “3” = strong staining. Scores for hnRNP A18 were then tabulated and expressed as a percentage of the respective cases analyzed.

### Ribonucleoprotein (RNP) immunoprecipitation

RNA bound to hnRNP A18 was immunoprecipitated as described in [12] [10] [6]. Briefly the cells were lysed with polysome lysis buffer (10mM Hepes, pH 7.0, 100mM KCl, 5mM MgCl_2_, 0.5% NP-40) and total lysates (1.5 mg) were incubated in the presence of EDTA (20 mM) with protein A-Sepharose CL-4B beads (Sigma) that had been pre-coated with 30 μg of either anti-hnRNP A18/CIRP (Sigma, cat: 121-135) or Normal rabbit anti-IgG1 (Calbiochem, Cat: NI01)). The beads were washed six times with NT2 buffer (50 mM Tris-HCl (pH 7.5), 150 mM NaCl, 1 mM MgCl_2_ and 0.05% NP-40) and then incubated with 100μl NT2 buffer containing 20 U RNase free DNase I for 15 min at 37°C. Bound proteins were then digested by adding 0.1% SDS and 0.5 mg/ml proteinase K and the reactions were continued for 15 min at 55°C. RNA was extracted by ethanol precipitation and used to perform RT-PCR. The PCR products were visualized after electrophoresis in 1% agarose gels.

Primers for RT-PCR were:

HIF-1α: 5′>AACCCATTCCTCACCCATCA<3′, 5′>TCCACCTCTTTTGGCAAGCA <3′,

GAPDH: 5′>ACATCAAGAAGGTGGTGAAGCAGG<3′, 5′>CCAGCAAGGATACTGAGAGCAAGAG <3′.

Where indicated the cells were exposed to CoCl_2_ (200 μM) for 2.5h prior to immunoprecipitation.

### mRNA stability assay

Cells were pre-treated with CoCl_2_ for 4 hours, then Actinomycin D (10 μg/ml) was added to inhibit transcription. RNA was collected at predetermined time points of 0h, 2h, 4h, 6h, and 8h by RNeasy Mini Kit (Qiagen), then reverse transcribed with the Reverse Transcription System (Promega). Resulting complementary DNA (cDNA) was combined with Real-Time TaqMan Gene Expression Assays primers for HIF-1α and GAPDH and then run on the Applied Biosystems 7300 Real-Time PCR System. Data was normalized relative to GAPDH and nonlinear regression analysis was used to determine half-life.

### Western blots

Human tissues were obtained from the University of Maryland Medical Center, Pathology Biorepository, Research Core. Rabbit polyclonal hnRNP A18 antibody was produced and purified in our laboratory and also purchased from Sigma (CIRP) and used at 1/500 dilution.

HIF-1α, mouse was from Cell Signaling Technologies and used at 1/1000 dilution. Actin mouse monoclonal antibody was from Abcam and used at 1/2000 dilutions. Secondary antibody and protein detection procedure were as described before [10].

### *De novo* protein synthesis

The Click-iT® Metabolic Labeling Reagents for Proteins (Invitrogen) kit was performed in the presence or absence of CoCl_2_, according to manufacturer's instructions. Briefly, cells were cultured in RPMI methionine-free media for 1 hour, then incubated in the same media with Click-iT® HPG (L-homopropargylglycine) for 4 hours. Proteins were extracted and the Click-iT® Protein Reaction Buffer Kit (Invitrogen) was used to label alkyne-tagged proteins with azide-biotin via a CuSO_4_ catalytic reaction. TRX antibody was incubated with the newly biotinylated protein lysate overnight, then protein A beads were added for 2 hours. Beads were washed 3 times and proteins were run on a SDS-PAGE gel. Newly synthesized proteins were detected by chemiluminescent with anti-Streptavidin-HRP antibody. Actin was used as loading control.

### Angiogenesis Proteome Profiler Array

Human Angiogenesis Proteome Profiler Array was performed as described by the manufacturer (R&D Systems Inc., Minneapolis, MN). Briefly, 300μg of proteins extracted from Lox scrambled and Lox shLentiA18 mice xenograft tumors were incubated with Array Buffer 4, Array Buffer 5, and Detection Antibody Cocktail at room temperature for 1 hour. Simultaneously, nitrocellulose membranes spotted in duplicate with capture antibodies to 55 specific angiogenesis-related human proteins were blocked in Array Buffer 7. After 1 hour, Array Buffer 7 was removed and sample/antibody mixtures were added to the membranes for overnight incubation. Membranes were washed with 1x Wash Buffer the following morning, incubated with Streptavidin-HRP, then detected with Chemi Reagent Mix on autoradiographic film. Positive signals were analyzed by densitometry and expressed as mean pixels density after subtracting background.

### Statistical analysis

Statistical analysis was performed on the relative (%) ratios of survival colonies expressing reduced levels of hnRNP A18 (shhnRNP A18) over wild type cells, on the weight of tumors stably transfected with shhnRNP A18 over tumor developing with the parent cell line and on HIF-1α relative levels of expression. All calculations including calculations for proliferation, invasion and migration, were performed by the Student t test. Probability values <0.05 are considered significant.

## SUPPLEMENTARY MATERIALS AND METHODS FIGURES


