# Supplementary material for: Heterogenous ribonucleoprotein A18 (hnRNP A18) promotes tumor growth by increasing protein translation of selected transcripts in cancer cells
Source: Oncotarget. 2016 Jan 25;7(9):10578–93. doi: 10.18632/oncotarget.7020 (PMC4891142; doi:10.18632/oncotarget.7020)
Supplement: Supplementary file 1 [file oncotarget-07-10578-s001.pdf]

# SUPPLEMENTARY MATERIALS AND METHODS

## Transfection

Lox-IMVI cells were transiently transfected with siRNA directed against HIF-1α (Thermo Fisher Scientific, Waltham, MA) using siPORT™ *NeoFX* Transfection Agent (Thermo Fisher Scientific, Waltham, MA) according to manufacturer's instructions. Briefly, HIF-1α siRNA and siPORT *NeoFX* were diluted separately in Opti-MEM and incubated for 10 min. Following incubation, the diluted transfection agent and siRNA were combined and allowed to incubate for another 10 min in order to allow transfection complexes to form. The mixture was then dispensed into 60mm plates (for Western blot analysis) or 96-well plates (for WST-1 proliferation assay) and Lox-IMVI cells were overlaid onto transfection complexes.

## Cell proliferation assay

Cell viability and proliferation were assessed using the Cell Proliferation Reagent WST-1 (Roche Diagnostics Corporation, Indianapolis, IN). Following transient transfection with HIF-1α siRNA, cells were incubated for 48 hours to allow for the down-regulation of HIF-1α. Cobalt chloride ($CoCl_2$) was then added and cells were allowed to incubate in a hypoxia chamber (0.5% $O_2$) for an additional 24 hours. Following $CoCl_2$ treatment, WST-1 reagent was then added to cells and absorbance was obtained by a multi-well ELISA plate reader.

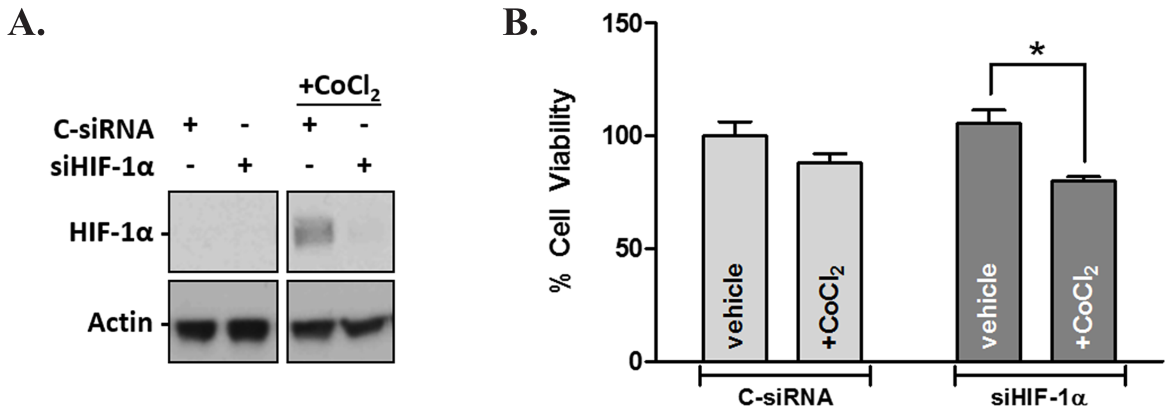


**Supplementary Figure S1: Down regulation of HIF-1α reduces cell viability in the presence of $CoCl_2$. A.** Western blot analysis of LOX-IMVI cells transiently transfected with scrambled siRNA (control, C-siRNA) or HIF-1α siRNA (siHIF-1α) and treated (+) or not (-) with 50 µM $CoCl_2$. The blots were hybridized with the indicated antibodies. **B.** Cell viability performed on LOX-IMVI cells transiently transfected with scrambled siRNA (control, C-siRNA) or HIF-1α siRNA (siHIF-1α) and treated (+) or (-) with 50 µM $CoCl_2$. WST-1 assays were performed as indicated in Material and Methods and viability is expressed as a percentage of the respective untreated samples. *= $p<0.05$

| Target | Sequence Alignment | Region | Accession |
| --- | --- | --- | --- |
| TRX | ttaaaacttgtaatttttttaattt-acaaaaatataaaatatgaaga | (560-606) | NM_003329 |
| ATR | tccatatatgtgaaatgaaattatgtaaaagaatatgttaataatcta | (8045-8092) | NM_001184 |
| EIF4G3 | tactct--tgaaatctttagagca--actttaaggcttgtaaatac-a | (5708-5750) | NM_003760 |
| EIF5A | tcccagggtggcgg-tggtggcagc-agtgatcctctgaacctgcaga | (708-753) | NM_001970 |
| EIF4E-BP2 | ttttag--tgtggtttattgagttc-agcagttctcatattctgttta | (3691-3735) | NM_004096 |
| EEF1A1 | tcagaac-tgtttgtttcaattggccatttaagtttagtagtaaaaga | (1507-1553) | NM_001402 |
| EEF1E1 | tgtctag-tgttgtcttcatcaaga-acagactatatactaattccca | (550-595) | NM_004280 |
| RPA | tccaggg-tgggatttcttgaggaagttacaaataagcttgttaca | (1383-1427) | NM_002946 |
| EIF3H | tgaactcttgaagtcacacc---agggcaactcttggaagaaat-a | (1117-1158) | NM_003756 |
| EIF4E-BP1 | taggttgatgtgcttgggaa---agctccctccccctccttcccca | (778-820) | NM_004095 |

**Supplementary Figure S2: NCBI Accession numbers and sequence alignment of a hnRNP A18 RNA recognition motif found in the indicated transcripts.** Invariant nucleotide [6] are in shaded boxes and the predicted positions of hnRNP A18 motif relative to the 3'UTR start site are indicated in parentheses.
